# Predicting Severe Disease and Critical Illness on Initial Diagnosis of COVID-19: Simple Triage Tools

**DOI:** 10.3389/fmed.2022.817549

**Published:** 2022-02-10

**Authors:** Lutfi Ali S. Kurban, Sharina AlDhaheri, Abdulbaset Elkkari, Ramzi Khashkhusha, Shaikha AlEissaee, Amna AlZaabi, Mohamed Ismail, Omran Bakoush

**Affiliations:** ^1^Department of Radiology, Tawam Hospital, Al Ain, United Arab Emirates; ^2^Department of Internal Medicine, Tawam Hospital, Al Ain, United Arab Emirates; ^3^Department of Internal Medicine, College of Medicine and Health Sciences, United Arab Emirates University, Al Ain, United Arab Emirates

**Keywords:** COVID-19, disease progression, critical illness, prediction model, prognosis

## Abstract

**Rationale:**

This study was conducted to develop, validate, and compare prediction models for severe disease and critical illness among symptomatic patients with confirmed COVID-19.

**Methods:**

For development cohort, 433 symptomatic patients diagnosed with COVID-19 between April 15th 2020 and June 30th, 2020 presented to Tawam Public Hospital, Abu Dhabi, United Arab Emirates were included in this study. Our cohort included both severe and non-severe patients as all cases were admitted for purpose of isolation as per hospital policy. We examined 19 potential predictors of severe disease and critical illness that were recorded at the time of initial assessment. Univariate and multivariate logistic regression analyses were used to construct predictive models. Discrimination was assessed by the area under the receiver operating characteristic curve (AUC). Calibration and goodness of fit of the models were assessed. A cohort of 213 patients assessed at another public hospital in the country during the same period was used to validate the models.

**Results:**

One hundred and eighty-six patients were classified as severe while the remaining 247 were categorized as non-severe. For prediction of progression to severe disease, the three independent predictive factors were age, serum lactate dehydrogenase (LDH) and serum albumin (ALA model). For progression to critical illness, the four independent predictive factors were age, serum LDH, kidney function (eGFR), and serum albumin (ALKA model). The AUC for the ALA and ALKA models were 0.88 (95% CI, 0.86–0.89) and 0.85 (95% CI, 0.83–0.86), respectively. Calibration of the two models showed good fit and the validation cohort showed excellent discrimination, with an AUC of 0.91 (95% CI, 0.83–0.99) for the ALA model and 0.89 (95% CI, 0.80–0.99) for the ALKA model. A free web-based risk calculator was developed.

**Conclusions:**

The ALA and ALKA predictive models were developed and validated based on simple, readily available clinical and laboratory tests assessed at presentation. These models may help frontline clinicians to triage patients for admission or discharge, as well as for early identification of patients at risk of developing critical illness.

## Introduction

Coronavirus disease 2019 (COVID-19) is a new respiratory infectious disease that was first reported in Wuhan, China and has subsequently spread worldwide. It is estimated that 80% of patients with COVID-19 have mild flu-like symptoms or no symptoms (1). However, up to a quarter of adult patients develop a severe respiratory illness that may progress to respiratory failure with high risk of mortality (2–5). This is causing a significant strain on health services worldwide.

Developing a reliable clinical tool to predict patient outcome at an early stage of the disease would greatly improve the management of patients and also ensure optimal utilization of health care resources. Moreover, early identification of patients with potential critical illness is crucial for early provision of the required supportive care and also appropriate selection for therapeutic trials. Furthermore, recognition of patients with potential non-severe disease is helpful to prompt discharge and alleviate the burden on the healthcare system (2, 3, 5). An ideal triage system in a pandemic setting should be quick, readily available, feasible, and affordable with the latter particularly relevant in resource-constraint setting. Various predictive factors have been evaluated to model disease outcomes in patients with COVID-19 (6–8), however, there are several limitations to the current literature. Firstly, most of these studies are from Chinese, European or American populations, with few or no reports from the Middle East. As the population age structure, socioeconomic status, and prevalence of underlying health comorbidities vary in different population groups, the proposed predicative factors may not be applicable to other populations. This study was conducted in the United Arab Emirates (UAE), which hosts over 200 nationalities, and about 88.5% of the population are expatriates and immigrants from other Middle Eastern and South Asian countries (9). Secondly, a recent critical appraisal and systemic analysis revealed that most published predictive models have significant selection bias and poorly defined predictors and outcomes, as well as lacking calibration and validation, making their generalizability and implementation across different settings and populations questionable (10, 11). Our predictive models were developed in accordance with the TRIPOD statement's recommendations with complete data on disease progression and clearly defined final outcomes (12). Finally, a recent review of the current screening and triage tools for COVID 19 concluded that almost all published validated triage tools rely on resource-intensive laboratory and imaging investigations, limiting their generalizability and utility in low-resource settings (13).

The aim of this study is to predict the risk of progression to severe disease and critical illness in symptomatic adult patients with confirmed COVID-19 infection based exclusively on simple and readily available baseline clinical parameters on initial diagnosis. We developed and validated a three- variable predictive model for severe disease and a four-variable model for critical illness and we compared their diagnostic accuracy with previously published predictive models. To the best of our best knowledge, this is the first study in the Middle East to develop validated predictive models for patients with COVID-19 infection to facilitate early prediction of severe disease and critical illness by measuring clinical and laboratory biomarkers at the time of presentation.

## Materials and Methods

### Participants and Study Design

From the early period of the epidemic, the governmental hospitals in Abu Dhabi adopted an admission policy for all cases of confirmed COVID-19 infection, including non-severe cases, to ensure isolation and also to limit potential viral spread in the community. To identify predictive factors for severe disease and critical illness, we started by reviewing the electronic health records (EHR) of all symptomatic adult patients (≥18 years) with suspected COVID-19 infection at Tawam Hospital, Abu Dhabi Emirate, UAE between 15th of April 2020 and 30th of June 2020. Of the 470 patients with suspected COVID-19, 37 patients had a negative swab and were excluded. Four hundred and thirty-three patients were included in our cohort and all had positive results on real time reverse transcription polymerase chain reaction (RT-PCR) assay of nasopharangeal swab specimens (AllplexTM 2019-nCoV Assay, Seegene Inc., Seoul, Republic of Korea). The results were validated using another cohort of 213 patients presented to another governmental hospital, Al Ain Hospital, Abu Dhabi, UAE during the same period and selected with similar inclusion/exclusion criteria. Our target population and outcomes were clearly defined, and we developed our predictive model using the recommendations and checklist of the TRIPOD statement (12). This study was approved by the Institutional Review Board and the Department of Health Ethical Committee, and written informed consent was waived.

### Data Collection

A team of respiratory clinicians reviewed and extracted the data from EHR using a standardized data collection tool. The generated data were checked independently by one clinician to ensure accuracy and consistency. Demographic data, comorbidities, symptoms and signs at presentation, laboratory biomarkers, and outcomes were collected and evaluated. We recorded information on respiratory support (high oxygen supplementation and mechanical ventilation), admission to intensive care unit (ICU), discharge, and death. Comorbidities were identified from the medical history section, previous visits, or at the time of diagnoses. Obesity was defined as a body mass index (BMI) ≥ 30 and was calculated as weight in kilograms divided by height in meters squared. The patients' identities were anonymized, and the data were password protected.

### Outcomes

We assessed two primary outcomes: severe disease and critical illness. The outcomes were evaluated longitudinally over the entire study period, not just at the time of the initial testing event. Final outcome of either discharge or death was obtained for all patients in our cohort and there were no censored data.

Severe disease was defined according to the Chinese management guideline for COVID-19 (14), version 6.0 as development of any of the following parameters during admission: respiratory rate ≥ 30/min, resting pulse oxygen saturation (SpO2) ≤ 93%, partial pressure of oxygen (PaO_2_)/fraction of inspired oxygen (FiO_2_) ≤ 300 mmHg (1 mmHg = 0.133 kPa).

Critical illness was defined as a composite event of death, septic shock or respiratory failure requiring high-level supplemental oxygen or mechanical ventilation (invasive or non-invasive). Almost all critically ill patients were admitted to the intensive care unit (ICU).

The criteria for discharge were absence of fever for at least 3 days, clinical remission of respiratory symptoms, and two negative RT-PCR swabs for COVID-19 obtained at least 24 h apart.

### Potential Predictors

We assessed 19 variables as potential predicators in our models based on the patients' demographic data, comorbidities, and laboratory biomarkers. Demographic variables included age, sex, and ethnicity/race. Comorbidities included obesity (according to the most recent BMI), hypertension, diabetes, cardiovascular disease, pulmonary disease (defined by chronic obstructive pulmonary disease or asthma), cerebrovascular disease (CVD), and chronic kidney disease (CKD). We assessed the laboratory biomarkers that have been reported to be commonly altered according to the recent recommendations in a systematic review and critical appraisal (10).

Laboratory biomarkers included the lymphocyte–neutrophil ratio (LN ratio), red cell distribution width (RDW), serum C-reactive protein (CRP), lactate dehydrogenase (LDH), albumin, D dimer and troponin T. The estimated glomerular filtration rate (eGFR) was calculated using CKD-EPI 2009 (15). We included only the first measurement obtained within 3 days of presentation.

### Statistical Analysis

The quantitative variables are reported as median and range. Categorical variables are presented as frequencies and proportions. To explore the association of risk factors with severe disease and critical illness, the Chi square test was used.

Univariate and multivariate logistic regression models were used to determine which predictive factors are associated with disease outcomes. All variables with *p* < 0.10 in the univariate analysis were included in the final multivariable models to determine if each variable is an independent predictor of outcomes. Multiple imputation procedure in IDM SPSS software was used to address the missing laboratory values for RDW (1.4% missing), CRP (2.1%), LDH (2.1%), D dimer (5.3%), eGFR (6.2%), albumin (14.1%), direct bilirubin (30.5%), and troponin T (36.3%). Data were assumed to be missing at random. Five imputations were performed and regression coefficients were pooled using Rubin's rules (16). All the other variables, including the outcome variables, were complete.

The regression analysis results are summarized as relative risk with 95% confidence interval (95% CI) and *p*-value. *P* ≤ 0.05 were considered statistically significant. All analyses were carried out using IBM SPSS software (version 27, Chicago, IL, USA).

### Statistical Prediction Models

The predictive models were constructed using predefined protocols according to the TRIPOD guidelines.

### Selection of Predictive Factors

We built our prediction models using a logistic regression model for the dichotomous outcomes of interest: disease severity and critical illness. Variables that strongly associated with the outcome of interest during stepwise multivariate analysis were selected for building the prediction model. Covariates that improved the performance of the model were included in the final model. The statistical assumptions of linearity for the model were met.

### Discrimination

Discrimination is the predictive accuracy of a model and is a measure of its ability to distinguish between those with and those without the outcome. This was assessed with concordance statistic (C statistic) by measuring the area under the receiver operating characteristic curve (AUC), which ranges from 0.5 to 1. AUC ≥ 0.7 was considered acceptable, whereas an AUC between 0.8 and 0.9 indicated excellent diagnostic accuracy.

### Calibration

Calibration refers to the agreement between the observed and predicted number of outcomes, and it measures the goodness of fit. The calibration was assessed by plotting the predicted risk of outcome on the x-axis against the observed risk of outcome on the y-axis. Further assessment of goodness of fit was performed by applying the Hosmer–Lemeshow test, which determines if the difference between the observed and predicted risks is significant or not. A *p* ≤ 0.05 was considered indicative of model lack of fit.

### Validation

To examine the prediction model's reproducibility and generalizability, we validated our predictive models on a validation cohort from Al-Ain hospital, Abu Dhabi, UAE. The predictive variables required to calculate risk score were collected from the validation cohort and the AUC was calculated.

## Results

### Characteristics of the Patients

**Figure 1:**
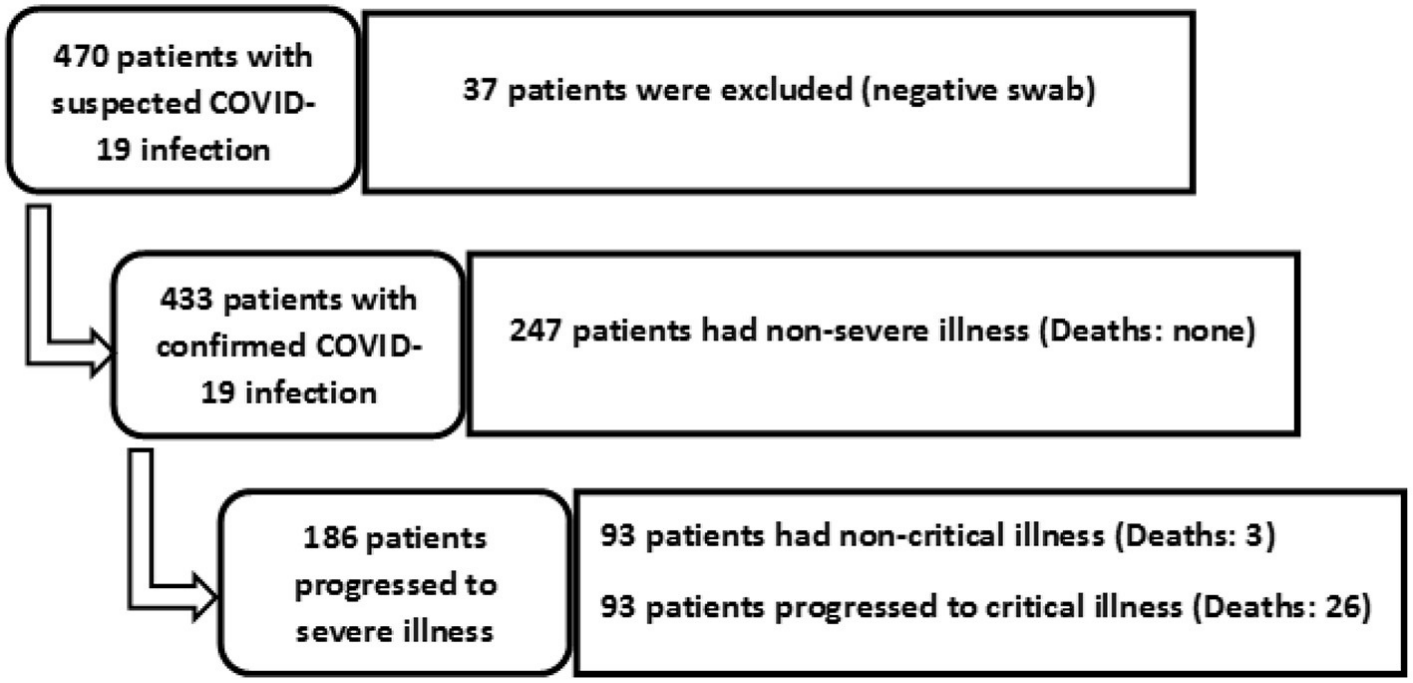
Flow diagram of patient selection, categorization, and outcome.

Of 470 patients triaged for suspected COVID-19 infection, 433 patients with a positive nasopharyngeal swab RT-PCR were included in the study. The median age was 48 years (range 18–90 years), and 99 patients (22%) were females. The disease was severe in 186 patients (43%). All 247 patients with non-severe disease recovered and were discharged. Of the 186 patients with severe disease, 93 patients developed critical illness, with 75 patients required mechanical ventilation. The other 18 patients received non-invasive ventilation support. The median time from admission to invasive mechanical ventilation was 5 days (range 1–21). The median time from mechanical ventilation to death was 7 days (range 1–29 days). Figure 1 illustrates the recruitment of participants and their final outcomes.

The most prevalent comorbidities were hypertension (34.6%), diabetes mellitus (33.7%), cardiovascular disease (11.3%) and CKD (8.8%). Non-Emirati patients represented 84.1% of the study sample, reflecting the national demographic composition. Asians were the most prevalent ethnic group (65.5%). Arabs were the second most prevalent ethnic group (32.1%) and Africans were the minority (2.4%). BMI was available for 363 patients, 129 (35%) of whom were obese with BMI ≥ 30. Patients with severe disease/ critical illness were more likely to have comorbidities than patients with non-severe illness. The most significant comorbidities were diabetes (40.3 vs. 28.7%), CKD (16.1 vs. 6.5%), and CVD (6.5 vs. 0.8%).

The most common clinical symptoms were fever (84.3%), cough (70.2%), shortness of breath (51.2%), fatigue (39.5%), sore throat (17.3%), and chest pain (17.1%). Median duration of symptoms was 4 days (range 1–15 days).

### Predictors of Severe Disease

In univariate analysis, severe disease was significantly associated with increasing age, being male, diabetes, CKD, and CVD, as well as with low LN ratio, serum albumin, and eGFR. Severe disease was also associated with high RDW, CRP, LDH, D-dimer, troponin T, and direct bilirubin. Severe disease was not associated with ethnic origin, hypertension, cardiovascular disease, pulmonary disease, or BMI.

In stepwise multivariate logistic regression analysis, severe disease was significantly associated with increasing age, male gender, and high RDW, LDH, CRP and D dimer, and with low LN ratio and albumin (Table 1).

**Table 1:** Univariate and multivariate regression analysis of predictive factors of severe COVID-19, critical illness, and in-hospital mortality.

**Demographics and comorbidities**	**Univariate analysis**				**Multivariate analysis**			
	**B**	**S.E**.	**Sig**.	**Exp(B) (95% CI)**	**B**	**S.E**.	**Sig**.	**Exp(B) (95% CI)**
**Disease severity**								
Age	0.033	0.007	0.001	1.033 (1.019–1.048)	0.030	0.008	0.001	1.03 (1.013–1.04)
Sex, Female/Male	0.701	0.245	0.004	2.016 (1.248–3.256)	0.950	0.267	0.001	2.58 (1.530–4.36)
Arabs National	0.056	0.269	0.834	1.058 (0.624–1.793)				
BMI	0.016	0.016	0.330	1.016 (0.984–1.049)				
Hypertension	0.231	0.203	0.256	1.260 (0.845–1.877)				
DM	0.516	0.205	0.012	1.675 (1.120–2.504)	0.063	0.242	0.794	1.06 (0.663–1.71)
CKD	1.021	0.326	0.002	2.776 (1.464–5.265)	0.666	0.375	0.076	1.94 (0.934–4.06)
CVA	2.134	0.770	0.006	8.448 (1.867–38.224)	1.420	0.809	0.079	4.13 (0.848–20.2)
CVD	0.552	0.305	0.07	1.737 (0.955–3.160)	0.108	0.349	0.757	0.89 (0.453–1.77)
Asthma/COPD	−0.614	0.541	0.256	0.541 (0.187–1.563)				
**Laboratory biomarkers**								
LN ratio	−2.370	0.197	0.001	0.093 (0.063–0.138)	−0.607	0.194	0.002	0.545 (0.372–0.797)
RDW	0.091	0.019	0.001	1.095 (1.055–1.136)	0.108	0.026	0.001	1.114 (1.059–1.172)
CRP	0.013	0.001	0.001	1.013 (1.012–1.015)	0.003	0.001	0.002	1.003 (1.001–1.004)
LDH	0.012	0.001	0.001	1.012 (1.011–1.013)	0.011	0.001	0.001	1.011 (1.010–1.012)
D dimer	0.326	0.032	0.001	1.386 (1.301–1.476)	0.088	0.033	0.007	1.092 (1.025–1.164)
Albumin	−0.217	0.011	0.001	0.805 (0.788–0.822)	−0.129	0.014	0.001	0.879 (0.854–0.904)
eGFR	−0.007	0.001	0.001	0.993 (0.990–0.995)	−0.001	0.002	0.711	0.999 (0.996–1.003)
Direct bilirubin	0.058	0.008	0.001	1.060 (1.044–1.077)	0.013	0.010	0.192	1.013 (0.993–1.034)
Troponin	0.001	0.001	0.041	1.000 (1.000–1.000)	0.001	0.001	0.722	1.000 (1.000–1.000)
**Critical Illness**								
Age	0.049	0.009	0.001	1.050 (1.032–1.067)	0.039	0.010	0.001	1.039 (1.020–1.060)
Sex, Female/Male	0.316	0.291	0.278	1.371 (0.775–2.425)				
Arabs National	−0.211	0.308	0.493	0.810 (0.443–1.481)				
BMI	0.012	0.019	0.525	1.012 (0.976–1.049)				
Hypertension	0.388	0.237	0.102	1.474 (0.926–2.347)				
DM	0.948	0.237	0.001	2.580 (1.621–4.104)	0.353	0.271	0.193	1.423 (0.837–2.421)
CKD/ESKD	1.147	0.324	0.001	3.148 (1.669–5.938)	0.443	0.378	0.241	1.557 (0.743–3.263)
CVA	1.927	0.571	0.001	6.869 (2.245–21.019)	1.032	0.612	0.091	2.808 (0.847–9.310)
CVD	0.611	0.330	0.064	1.842 (0.966–3.515)	−0.214	0.383	0.577	0.808 (0.381–1.712)
Asthma/COPD	−0.295	0.647	0.648	0.744 (0.209–2.645)				
**Laboratory biomarkers**								
LN ratio	−3.017	0.294	0.001	0.049 (0.028–0.087)	−0.746	0.267	0.005	0.474 (0.281–0.800)
RDW	0.191	0.021	0.001	1.210 (1.163–1.260)	0.210	0.026	0.001	1.234 (1.173–1.297)
CRP	0.010	0.001	0.001	1.010 (1.009–1.012)	0.004	0.001	0.001	1.004 (1.003–1.006)
LDH	0.006	0.001	0.001	1.006 (1.005–1.007)	0.005	0.001	0.001	1.005 (1.004–1.005)
D dimer	0.293	0.028	0.001	1.340 (1.269–1.414)	0.107	0.026	0.001	1.112 (1.057–1.171)
Albumin	−0.203	0.011	0.001	0.817 (0.799–0.835)	−0.095	0.015	0.001	0.910 (0.884–0.936)
eGFR	−0.015	0.002	0.001	0.985 (0.982–0.988)	−0.008	0.002	0.001	0.992 (0.988–0.995)
Direct bilirubin	0.063	0.009	0.001	1.065 (1.048–1.084)	0.038	0.010	0.001	1.039 (1.018–1.060)
Troponin	0.001	0.001	0.169	1.000 (1.000–1.000)				

### Predictors of Critical Illness

In univariate analysis, critical illness was significantly associated with older age, diabetes, CKD and CVD, with lower LN ratio, albumin and eGFR, and with high RDW, CRP, LDH, D-Dimer, and direct bilirubin. Critical illness was not associated with gender, ethnic origin, hypertension, CVD, pulmonary disease, BMI, or serum troponin T.

In stepwise multivariate logistic regression analysis, critical illness was significantly associated with older age, being male, and high RDW, LDH, CRP, D dimer, and direct bilirubin, as well as with low LN ratio, albumin, and eGFR (Table 1).

### Prediction Models of Outcomes

#### Prediction Model for Severe Disease

**Figure 2:**
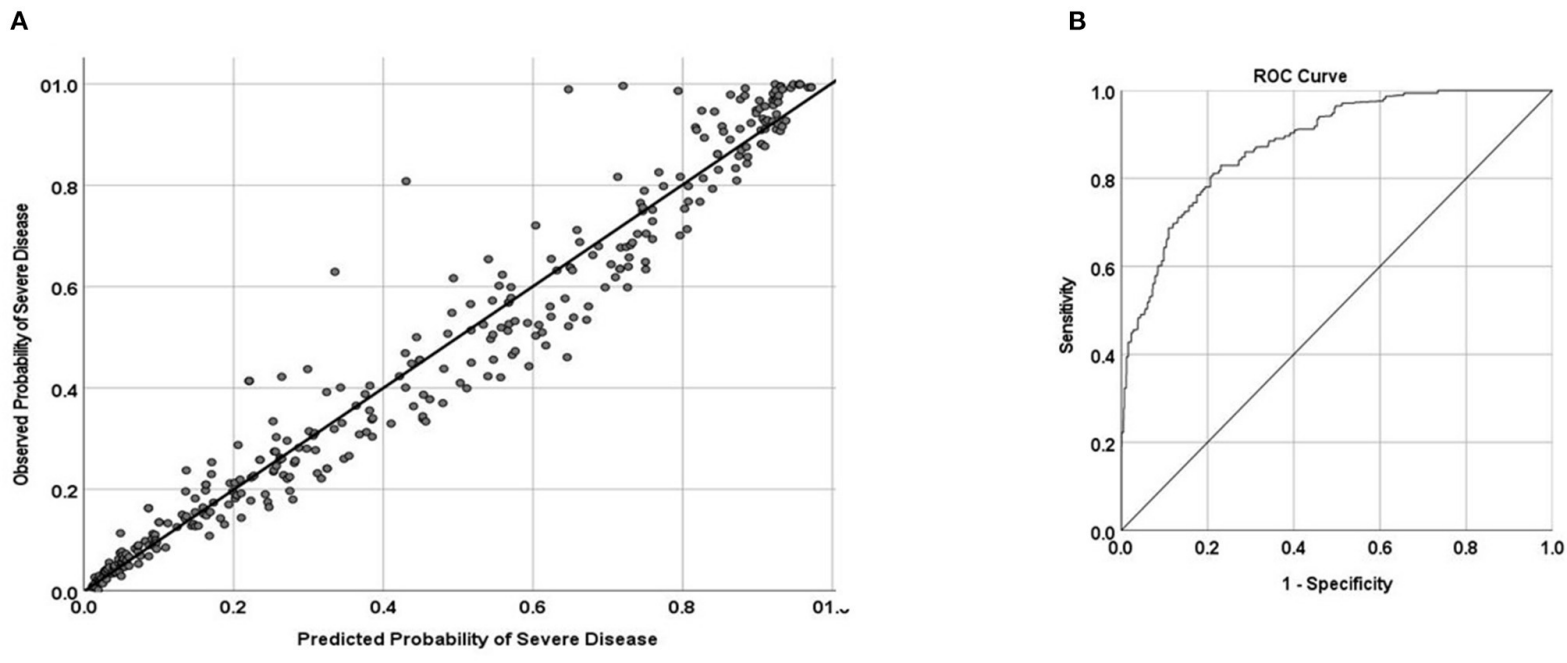
**(A)** Calibration and **(B)** area under the receiver operating characteristic curve (AUC) of predicting severe disease in patients with COVID-19 infection. AUC = 0.881 (95% CI, 0.868–0.893).

Three variables at presentation showed the best discriminating ability in predicting severe disease in patients with COVID-19 infection, namely, age, serum LDH, and serum albumin (ALA model). These three variables had an AUC of 0.881 (95% CI, 0.868–0.893), indicating excellent discrimination (Figure 2). The calibration plot indicated that the predicted probabilities matched the actual probabilities, suggesting very good calibration. The *p*-value for the Hosmer-Lemeshow test was 0.375, indicative of good fit.

#### Prediction Model for Critical Illness

**Figure 3:**
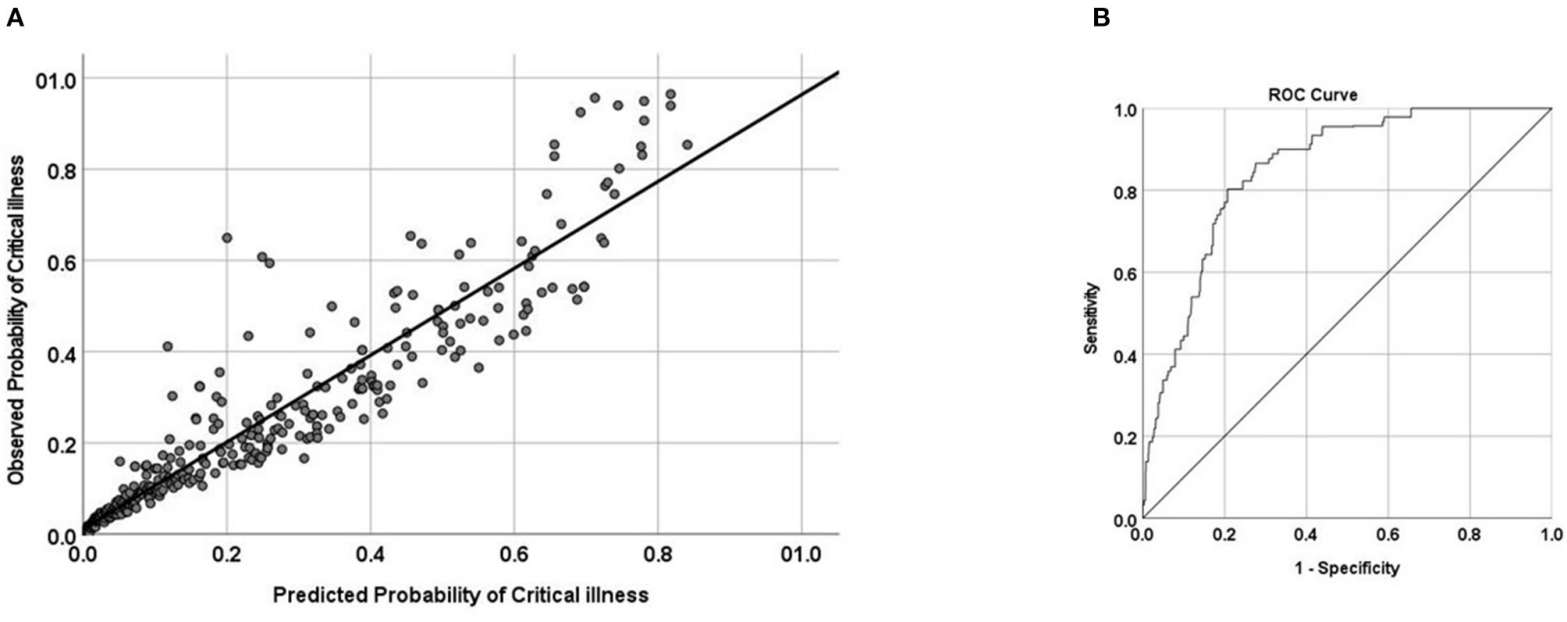
**(A)** Calibration and **(B)** area under the receiver operating characteristic curve (AUC) of predicting critical illness in patients with COVID-19 infection. AUC = 0.851 (95% CI, 0.835–0.867).

In this model, four variables were selected to predict critical COVID-19 illness at presentation. These factors, age, serum LDH, kidney function (eGFR) and serum albumin (ALKA predictive model), showed accurate discrimination, with an AUC of 0.851 (95% CI, 0.835–0.867, Figure 3). The calibration plot showed that predicted probabilities were very close to the observed probabilities, consistent with very good calibration. The *p*-value for the Hosmer-Lemeshow test was insignificant at 0.118, in keeping with good fit.

### Construction of the Risk Score and Web-Based Calculator

Equations for predicting the risks of severe disease and critical illness in individual patients were constructed based on the fitted risk models. Web-based calculators are available online (https://covidriskscore.000webhostapp.com/).

The prediction equation to assess an individual patient's severe disease risk score = – 0.96 + 0.03 (age, years) + 0.011 (LDH, U/L) – 0.13 (albumin, g/L).

The prediction equation to estimate the critical illness risk score = – 1.69 + 0.039 (age, years) + 0.005 (LDH, U/L) – 0.095 (albumin, g/L) – 0.008 (eGFR, ml/min/1.73 m^2^).

### Validation

The validation cohort included 213 patients with median age of 42 years (range 18–82 years), 38 were females (17.8%). Sixteen patients developed severe disease and subsequently progressed to critical illness (7.5%). Of the 16 patients who developed critical illness, 3 patients died (18.7%).

The most prevalent comorbidities were hypertension (22.5%), diabetes mellitus (21.1%), cardiovascular disease (3.7%), and CKD (2%).

**Figure 4:**
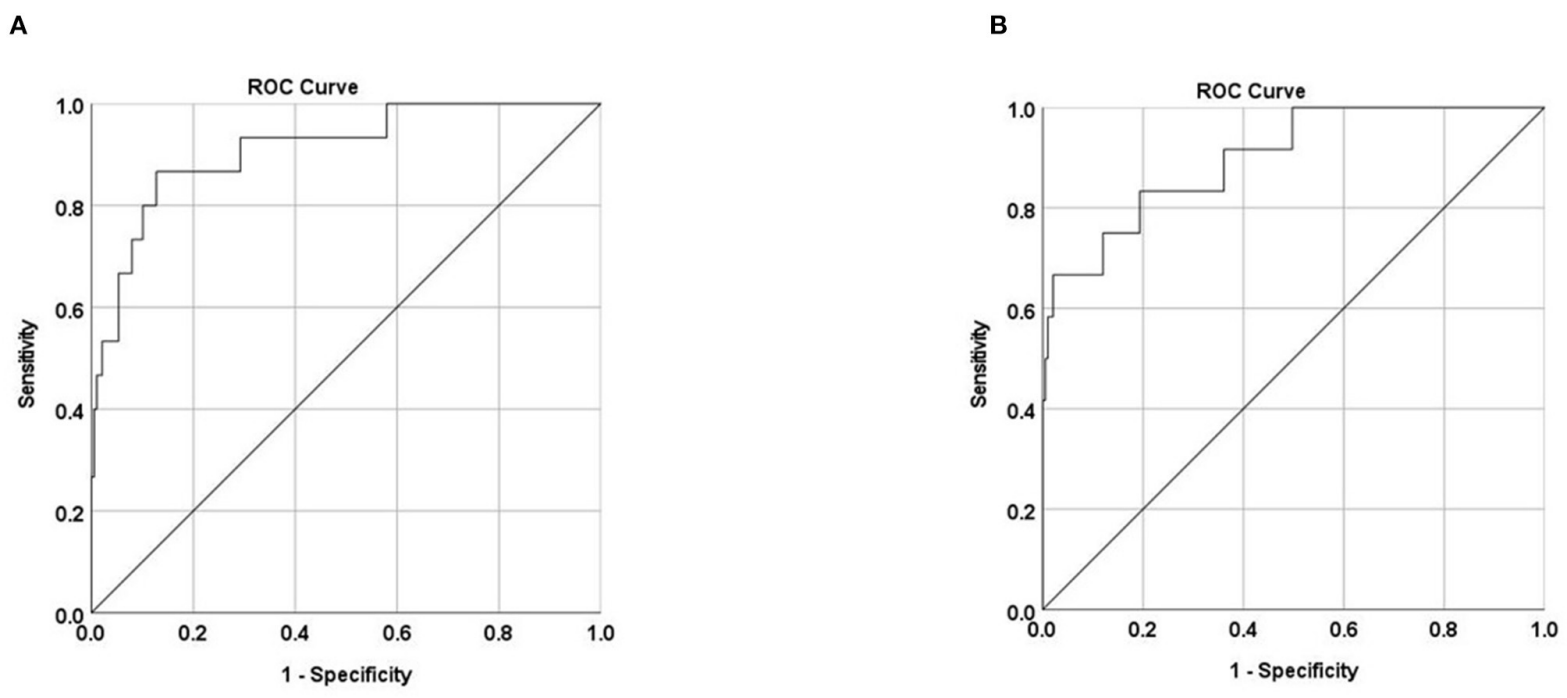
Area under the receiver operating characteristic curve (AUC) of predicting severe disease **(A)** and critical illness **(B)** in the validation cohort. AUC = 0.911 (95% CI, 0.832–0.990) and 0.899 (95% CI, 0.806–0.992), respectively.

The accuracy of the models' prediction of disease severity and critical illness were comparable to the development cohort, with an AUC of 0.911 (95% CI, 0.832–0.990) and 0.899 (95% CI, 0.806–0.992), respectively, indicating excellent discrimination (Figure 4).

### Comparison With Previously Published Models

Our ALA predictive model showed a high discriminatory performance (AUC, 0.88, 95% CI, 0.868–0.893) which outperformed the exiting HNC-LL score model (AUC, 0.85, 95% CI, 0.82–0.89) and the NLR model (AUC, 0.71, 95% CI, 0.659 to −0.759).

Similarly, the ALKA predictive model demonstrated a higher diagnostic accuracy (AUC, 0.851, 95% CI, 0.835–0.867) compared to the existing predictive model of critical illness published by Zhang et al. (AUC, 0.74, 95% CI, 0.66 to −0.82).

## Discussion

We developed and validated models to predict disease outcomes in adult patients with confirmed COVID-19 infection. As the clinical course of the disease is unpredictable, COVID-19 infection continues to pose serious challenges to the healthcare systems globally. Identifying patients who are at risk of developing severe disease will enable informed decisions about admission and discharge as well as proper healthcare resource utilization. Furthermore, early recognition of patients at risk of critical illness and possibly death will improve patient monitoring and timely initiation of the required level of care and support. One of our models' key features is that they utilize simple, readily available clinical parameters that can be assessed at the time of presentation. The development of our predictive models in a unique cohort of patients with both severe and non-severe illness at the time of initial presentation is another major strength.

We found that increasing age is a strong predictor of severe disease and critical illness. During aging, major changes occur in the immune system, collectively described immunosenescence, a condition characterized by declining immune functions (17). These changes include T-cell and B-cell dysfunction as well as excess production of type 2 cytokines, which could lead to uncontrolled viral replication, proinflammatory responses, and poor outcome (18).

The median age of our cohort was 48 years, which is lower than reported in other studies of American, European, and Chinese populations, reflecting demographic differences between these populations (19–22).

In our cohort, elevated serum LDH and low serum albumin at presentation were strong independent predictors of severe disease and critical illness. In patients with severe infection, elevated levels of LDH are attributed to the release of the intracellular content when the integrity of the cell membrane is compromised, such as in virally induced pulmonary damage. Additionally, LDH levels can be elevated in patients due to cytokine-mediated multiple organ injury (23, 24). In a pooled analysis of 1,532 patients with COVID-19 infection, elevated LDH levels were associated with a six-fold increase in the probability of developing severe disease and a 16-fold increase in the probability of death (24). Similarly, Muhammad et al. reported increased in-hospital mortality in COVID-19 patients with elevated LDH (25).

There are a few hypotheses regarding the cause of hypoalbuminemia in patients with severe COVID-19 infections. First, hypoalbuminemia is a recognized biomarker of acute and chronic inflammation. Moreover, essential amino acid consumption due to viral replication, transcriptional inhibition and albumin clearance might play a role in albumin depletion. Furthermore, reduced albumin synthesis by the liver and increased microvascular permeability and consequent redistribution of albumin into extravascular compartments have also been suggested to contribute to hypoalbuminemia (26). Albumin has strong anti-inflammatory, antioxidant and anticoagulation properties, so hypoalbuminemia can have prothrombotic effects with an increased risk of arterial and venous thrombosis in different clinical settings (27, 28).

We showed that eGFR on presentation was an independent predictor of critical illness in patients with COVID-19. It has been reported that expression of angiotensin-converting enzyme 2 (ACE2), the major cell entry receptor for SARS-CoV-2, is almost 100 times higher in the kidneys than in the pulmonary parenchyma (29). Acute kidney disease in patients with COVID-19 is caused by several factors, including direct cytopathic effects on kidney tissue, endothelial damage, deposition of immune complexes, and virus-induced cytokines (30). Several studies have shown higher mortality in patients with CKD. Cheng et al. reported that during hospitalization of patients with COVID-19, there is a high prevalence of kidney disease on admission, and that the development of acute kidney injury is frequent and is associated with in-hospital mortality. Guan et al. found that mortality and ICU admission rates of patients with raised serum creatinine were higher than in patients with normal serum creatinine level (9.6 vs. 1%). Lim et al. reported that the development of acute kidney injury is more strongly associated with severe clinical outcomes and mortality in patients with chronic kidney disease (20, 30, 31).

### Predictive Model of Severe Disease (ALA Model)

To predict severe disease at presentation in adult patients with COVID-19 infection, we developed a predictive model using three readily available clinical parameters (age, serum LDH, and albumin).

Our cohort included all symptomatic patients with both severe and non-severe COVID-19, as early on in the pandemic, the initial strategy in the country was to hospital isolate all confirmed positive cases to control the spread of the disease. All patients in our cohort who were predicted to have a course of non-severe illness at initial presentation were discharged without developing critical illness. Therefore, the ALA predictive model can be a useful tool for triaging patients with COVID-19 infection for admission or discharge.

The utility of the previously published models on predicting the risk of progression to severe COVID-19 and early identification of patients requiring hospital admission is limited (32–35). The COVID-19 vulnerability index model (CV19) has a serious limitation, as it was developed using proxy events and outcomes from non-COVID-19 pneumonia patients (32). The other predictive model developed by Meng et al. relies on subjective clinical symptoms such as breathlessness and serum biomarkers that are not readily available in the emergency department setting, e.g., serum interleukin 6 (35). We compared the accuracy of our ALA predictive model with other existing predictive models of severe disease. We applied the corresponding variables of the HNC-LL score model, hypertension, neutrophil count, serum CRP, lymphocyte count, and serum LDH, developed by Xiao et al. and the neutrophil-to-lymphocyte ratio (NLR) model developed by Liu et al. to our cohort (33, 34). We found that the discriminatory performance was lower for the HNC-LL model and the NLR model compared to our ALA predictive model. Noteworthy is that the HNC-LL and NLR models have been shown to outperform the CURB-65 and MuLBSTA scoring systems (33, 34).

### Predictive Model of Critical Illness (ALKA Model)

In this model, we developed a scoring system to predict critical illness using four readily available parameters: age, serum LDH, kidney function (eGFR), and serum albumin. We would like to emphasize the importance of the timing of measurement in this model, as we focused exclusively on baseline assessment of the predictive variables at presentation. Therefore, this model could become an important tool for clinicians to identify patients, at or soon after admission, who are at risk of developing a critical illness. This is of paramount value in providing early high-level support and early intervention.

Several previous studies have reported risk prediction models of critical illness and in-hospital mortality based on demographic data, clinical findings and laboratory biomarkers assessed at the time of hospital admission or following transfer to ICU. Most of the studies have been from Chinese, European or American populations, with few reports from the Middle East (10, 36–43).

The previously reported predictive models have several limitations. Firstly, up to 10 predictive variables were employed, and some studies included subjective symptoms or laboratory biomarkers that are not readily available (10, 33–35, 39). In our model, we assessed the laboratory markers that have been reported to be commonly altered according to recent recommendations based on systematic review and critical appraisal (10). The predictive variables we selected are simple, readily available, and can be accurately measured.

Secondly, some studies measured their predictors at an inappropriate time, which may have influenced the outcomes (8, 19, 44, 45). Measuring variables not at presentation but later at their peak during ICU admission may lead to a look-ahead bias. As mentioned above, our predictive parameters are part of the baseline assessment at presentation, and the models can provide an early score of critical illness risk while avoiding look-ahead bias.

Thirdly, study participants were often excluded because they did not develop the outcome at the end of the study, which means that the final outcome was not determined. This generates a highly selected study sample (46). In our cohort, the end points and the outcome measures were clearly defined, and all our patients had a final outcome of either discharge or progression to critical illness.

Finally, some of the previously published predictive models lacked calibration and validation. Calibration, which measures the goodness of fit, refers to the agreement between the observed and predicted outcomes. Validation refers to the process of confirming that the model actually achieves its intended purpose, and it ensures the model's reproducibility. We strictly followed TRIPOD guidelines, including discrimination, calibration and validation of the predictive models. Our models showed good fit and were validated with a separate validation cohort from another governmental hospital to ensure generalizability to all the UAE population, and probably to other nearby countries in the region.

We compared the discriminatory power of our ALKA model with the predictive model published by Zhang et al. which share two common predictive variables, age, and serum LDH with our ALKA model (41). Applying Zhang et al. model's predictive variables to our cohort showed a lower discriminatory power compared to our ALKA model. We were unable to evaluate the discriminatory performance of other previously published predictive models that utilized computed tomography findings, large numbers of parameters, or several uncommonly measured clinical and laboratory biomarkers.

Discussing prognostic triage tool for COVID 19 is not complete without referring to artificial intelligence (AI). There is no doubt that AI has the potential to transform how health care is delivered and it is becoming a more widely tested tool for emergency room triage including patients with COVID-19 infection. Many studies have investigated AI applications for diagnostic and prognostic triage of COVID 19 patients during the pandemic. However, the science behind the AI algorithm is deep and highly complex and it has the potential to suffer from a host of shortcomings, including bias and inapplicability outside of the training domain (47). A recent review of over 400 diagnostic and prognostic AI triage tools for COVID 19 infection highlighted serious methodological and reporting flaws that jeopardize reproducibility, generalizability, and usability for clinical practice (48). The utility of the best performing machine learning-based algorithm model was limited by using large number of unreadily available clinical parameters rendering it impractical in resource-constrained settings (49).

Our study has several limitations. Firstly, it was retrospective and all the data were collected from case records. Therefore, important information such as history of comorbidities might have been missed and some laboratory parameters were not available. To account for missing data, we used multiple imputations. Secondly, we limited our cohort to symptomatic patients with confirmed COVID-19 at presentation, potentially excluding patients with milder illness who did not seek medical advice. Thirdly, the number of patients included in the analysis was small, which may limit the interpretation of our model. Larger prospective studies are required to confirm our findings. Finally, predictive models need to be externally validated in other datasets to determine whether these models are generalizable to other cohorts outside the UAE. Given that our study was done early in the pandemic and the cohort was almost entirely comprised of unvaccinated patients, it would be interesting to test the ALA and ALKA models in vaccinated patients with breakthrough COVID-19 infection, to assess their validity.

## Conclusions

Age, serum LDH and serum albumin are independent predictors of severe disease in patients with COVID-19 infection. In addition to these three predictor factors, eGFR is another strong independent predictor of critical illness. We developed and validated two simple, accurate predictive models (ALA and ALKA) to stratify patients at presentation into non-severe, severe or critical illness. The ALA scoring system may provide frontline clinicians with a useful tool to triage patients for admission and discharge. The ALKA scoring system may allow early identification of patients at risk of developing critical illness and therefore may enable very close monitoring and early active treatment. The predictive parameters in these two models are simple and readily available and can be easily incorporated into EHR as a clinical decision support tool. External validation of these two predictive models using a larger cohort is suggested.

## Data Availability Statement

The raw data supporting the conclusions of this article will be made available by the authors, without undue reservation.

## Ethics Statement

The studies involving human participants were reviewed and approved by the Institutional Review Board and the Department of Health Ethical Committee, Abu Dhabi, UAE. Written informed consent for participation was not required for this study in accordance with the national legislation and the institutional requirements.

## Author Contributions

LK, SAlD, AE, RK, SAlE, AA, MI, and OB contributed to the design and implementation of the research. LK and OB contributed to the analysis of the results and to the writing of the manuscript. All authors discussed the results, commented on the manuscript, contributed to the article, and approved the submitted version.

## Funding

This work was supported by College of Medicine and Health Sciences, UAEU.

## Conflict of Interest

The authors declare that the research was conducted in the absence of any commercial or financial relationships that could be construed as a potential conflict of interest.

## Publisher's Note

All claims expressed in this article are solely those of the authors and do not necessarily represent those of their affiliated organizations, or those of the publisher, the editors and the reviewers. Any product that may be evaluated in this article, or claim that may be made by its manufacturer, is not guaranteed or endorsed by the publisher.
